# Supplementary material for: Comparative neuroanatomy of the lumbosacral spinal cord of the rat, cat, pig, monkey, and human
Source: Sci Rep. 2021 Jan 21;11:1955. doi: 10.1038/s41598-021-81371-9 (PMC7820487; doi:10.1038/s41598-021-81371-9)
Supplement: Supplementary file 1 — Supplementary Information. [file 41598_2021_81371_MOESM1_ESM.docx]

**Supplementary Material for**

Comparative Neuroanatomy of the Lumbosacral Spinal Cord of the Rat, Cat, Pig, Monkey, and Human

Amirali Toossi, Bradley Bergin, Maedeh Marefatallah, Behdad Parhizi, Neil Tyreman, Dirk G. Everaert, Sabereh Rezaei, Peter Seres, J. Christopher Gatenby, Steve I. Perlmutter, Vivian K. Mushahwar

**This file includes:**

Supplementary Table S1

Supplementary Figs. S1 to S7

**Corresponding Author:**

Vivian K. Mushahwar, PhD

5-005 Katz Building

University of Alberta,

Edmonton, AB, Canada

T6G 2E1

Phone: (780) 492-4519

Email: vivian.mushahwar@ualberta.ca

**Table S1.** Segmental ranges and length of the lumbosacral enlargement in individual samples

| **Sample ID** | **Species** | **Spinal Cord Levels of the Lumbosacral Enlargement** | **Length of the lumbosacral enlargement (mm)** |
| --- | --- | --- | --- |
| R1 | Rat | L2-S1 | 17 |
| R2 | Rat | L3-L6 | 12 |
| R3 | Rat | L3-S1 | 12 |
| R4 | Rat | L3-L6 | 10 |
| R5 | Rat | L3-S1 | 12 |
| R6 | Rat | L3-S1 | 11 |
| C1 | Cat | L4-S1 | 35 |
| C2 | Cat | L4-S2 | 37 |
| C3 | Cat | L4-S1 | 33 |
| C4 | Cat | L4-S1 | 33 |
| C5 | Cat | L4-S1 | 34 |
| C6 | Cat | L4-S2 | 34 |
| P1 | Pig | L3-S2 | 69 |
| P2 | Pig | L3-S1 | 76 |
| P3 | Pig | L3-S2 | 67 |
| P4 | Pig | L3-S1 | 60 |
| P5 | Pig | L3-S1 | 62 |
| P6 | Pig | L3-S1 | 66 |
| M1 | Monkey | L2-L7 | 31 |
| M2 | Monkey | L3-S1 | 32 |
| M3 | Monkey | L3-L7 | 30 |
| M4 | Monkey | L3-S1 | 38 |
| M5 | Monkey | L2-L7 | 33 |
| M6 | Monkey | L2-S2 | 45 |
| H1 | Human | T12-S1 | 50 |
| H2 | Human | T12-S2 | 64 |
| H3 | Human | T12-S2 | 56 |
| H4 | Human | L1-S1 | 57 |
| H5 | Human | L1-S1 | 56 |
| H6 | Human | L2-S2 | 63 |

**
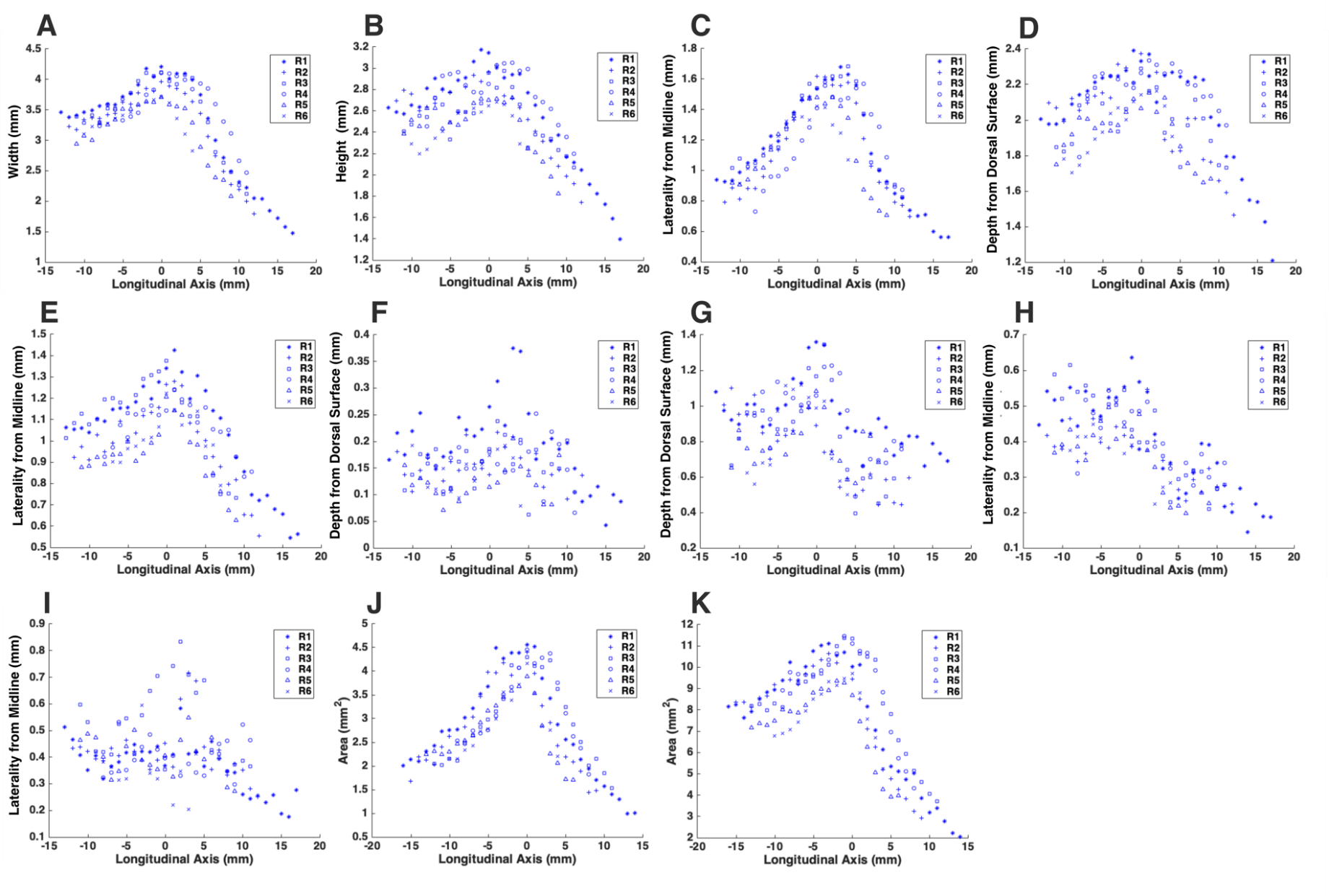
**

**Fig. S1.** Cross-sectional dimensions of the spinal cord and locations of the ventral and dorsal horns of rat cords. Presented data are based on n=6 specimens per species. Different symbols represent different specimens within a species. Measurements are of the parameters shown in Fig. 7 across the length of the lumbosacral cord. All curves have been translated across the x-axis such that the cord’s PCS (where d1 is maximum) is at the origin (x=0). A) d1. B) d2. C) d3. D) d4. E) d5. F) d6. G) d7. H) d8. I) d9. J) a1. K) a2.


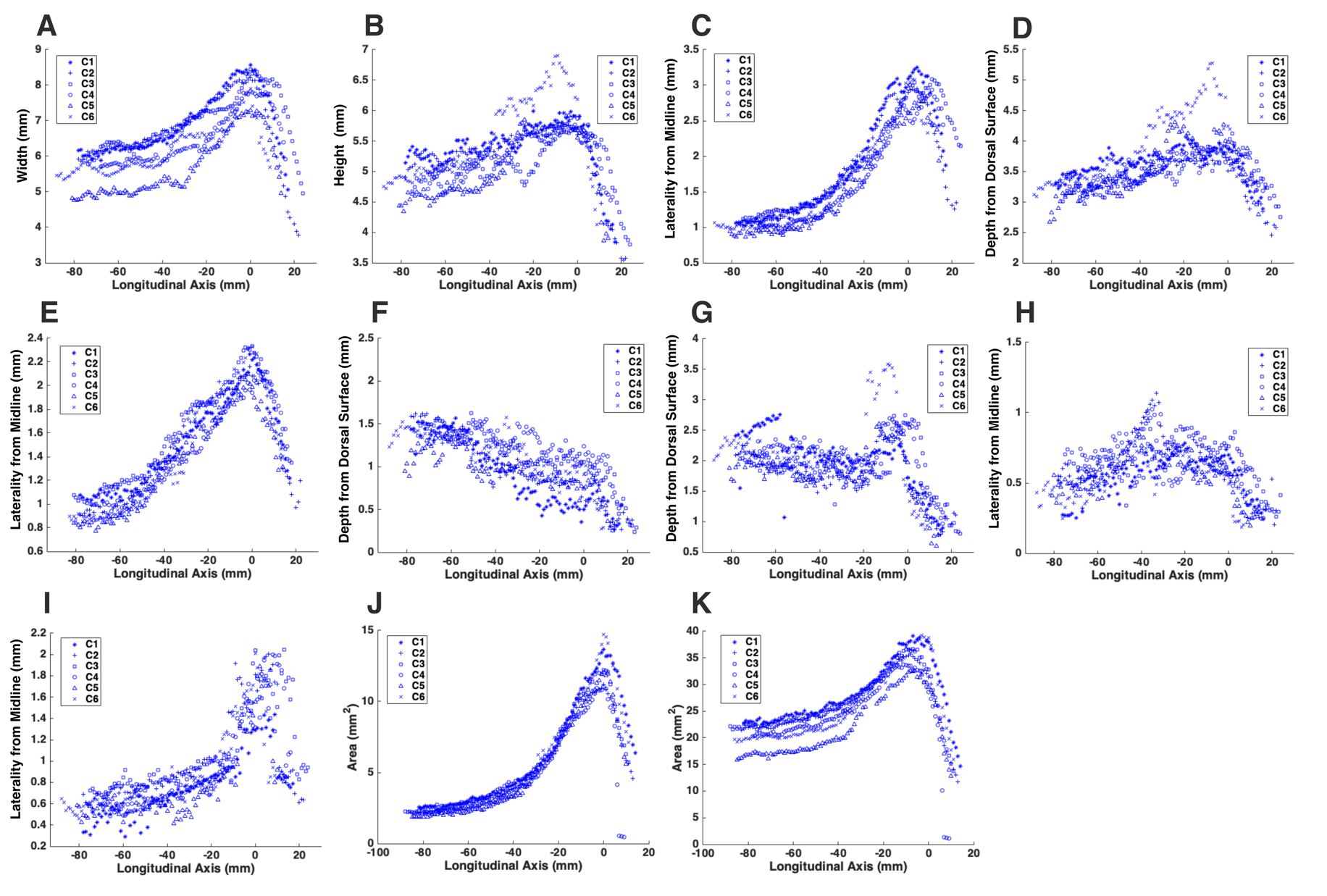


**Fig. S2.** Cross-sectional dimensions of the spinal cord and locations of the ventral and dorsal horns of cat cords. Presented data are based on n=6 specimens per species. Different symbols represent different specimens within a species. Measurements are of the parameters shown in Fig. 7 across the length of the lumbosacral cord. All curves have been translated across the x-axis such that the cord’s PCS (where d1 is maximum) is at the origin (x=0). A) d1. B) d2. C) d3. D) d4. E) d5. F) d6. G) d7. H) d8. I) d9. J) a1. K) a2.
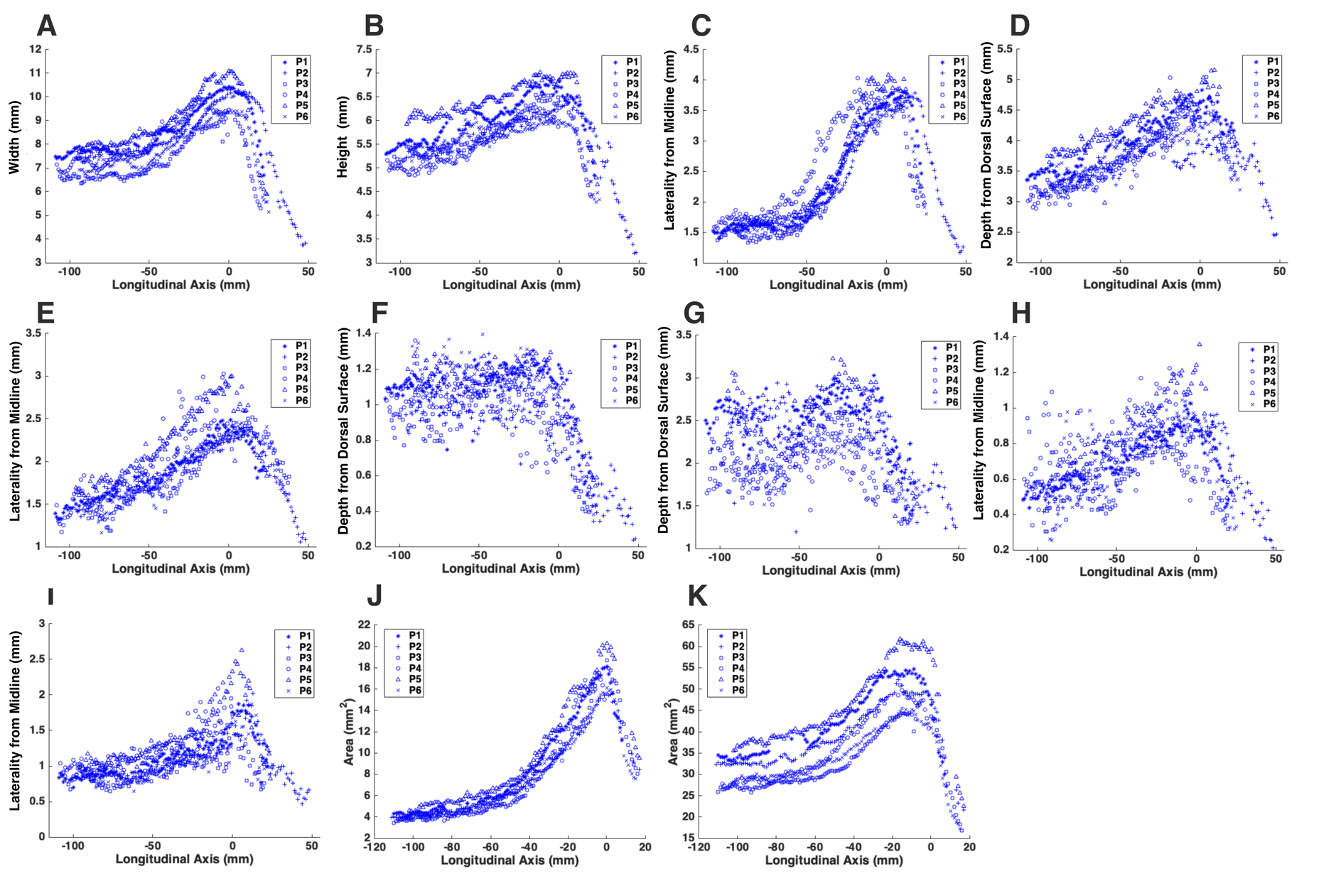


**Fig. S3.** Cross-sectional dimensions of the spinal cord and locations of the ventral and dorsal horns of pig cords. Presented data are based on n=6 specimens per species. Different symbols represent different specimens within a species. Measurements are of the parameters shown in Fig. 7 across the length of the lumbosacral cord. All curves have been translated across the x-axis such that the cord’s PCS (where d1 is maximum) is at the origin (x=0). A) d1. B) d2. C) d3. D) d4. E) d5. F) d6. G) d7. H) d8. I) d9. J) a1. K) a2.

**
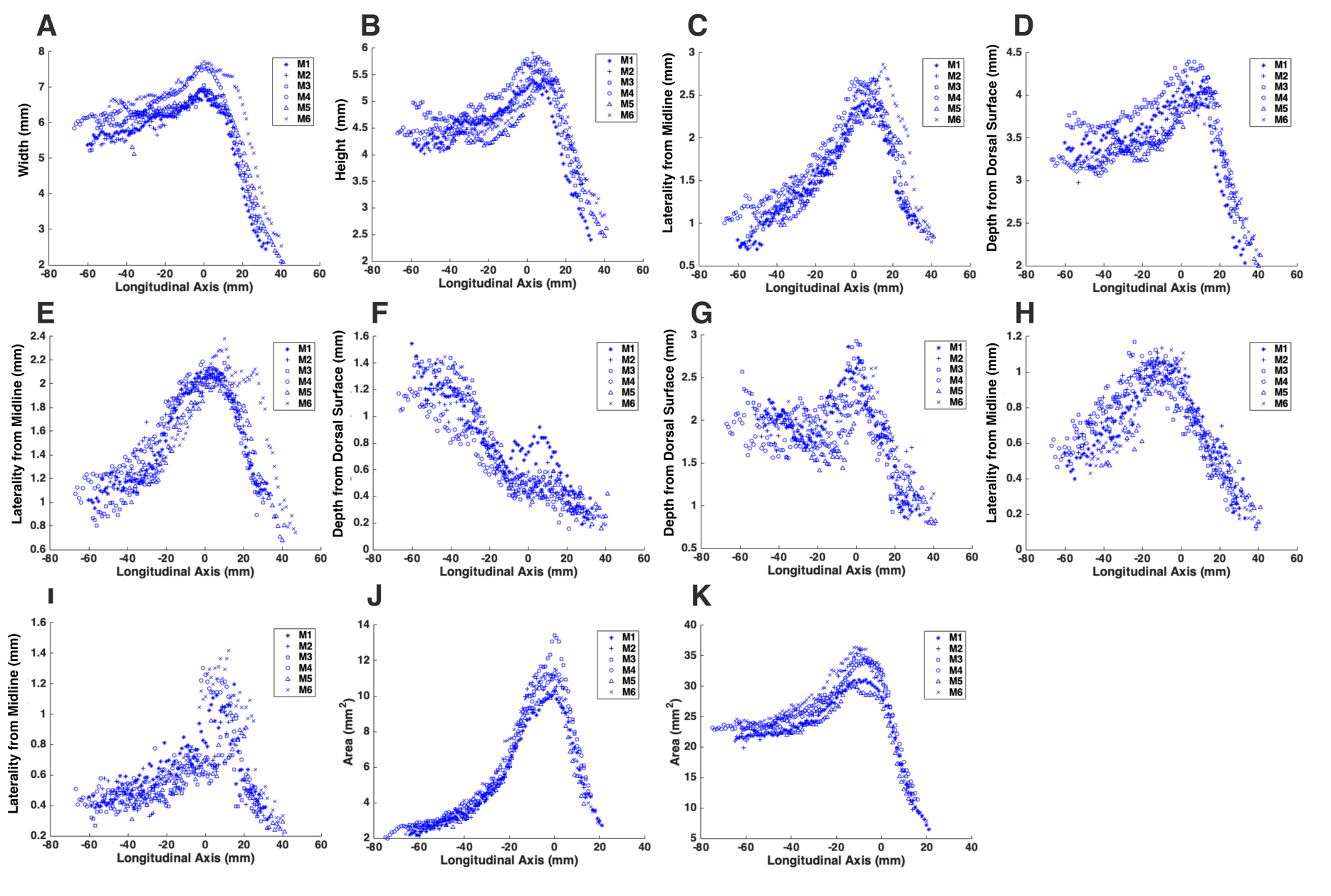
**

**Fig. S4.** Cross-sectional dimensions of the spinal cord and locations of the ventral and dorsal horns of monkey cords. Presented data are based on n=6 specimens per species. Different symbols represent different specimens within a species. Measurements are of the parameters shown in Fig. 7 across the length of the lumbosacral cord. All curves have been translated across the x-axis such that the cord’s PCS (where d1 is maximum) is at the origin (x=0). A) d1. B) d2. C) d3. D) d4. E) d5. F) d6. G) d7. H) d8. I) d9. J) a1. K) a2.


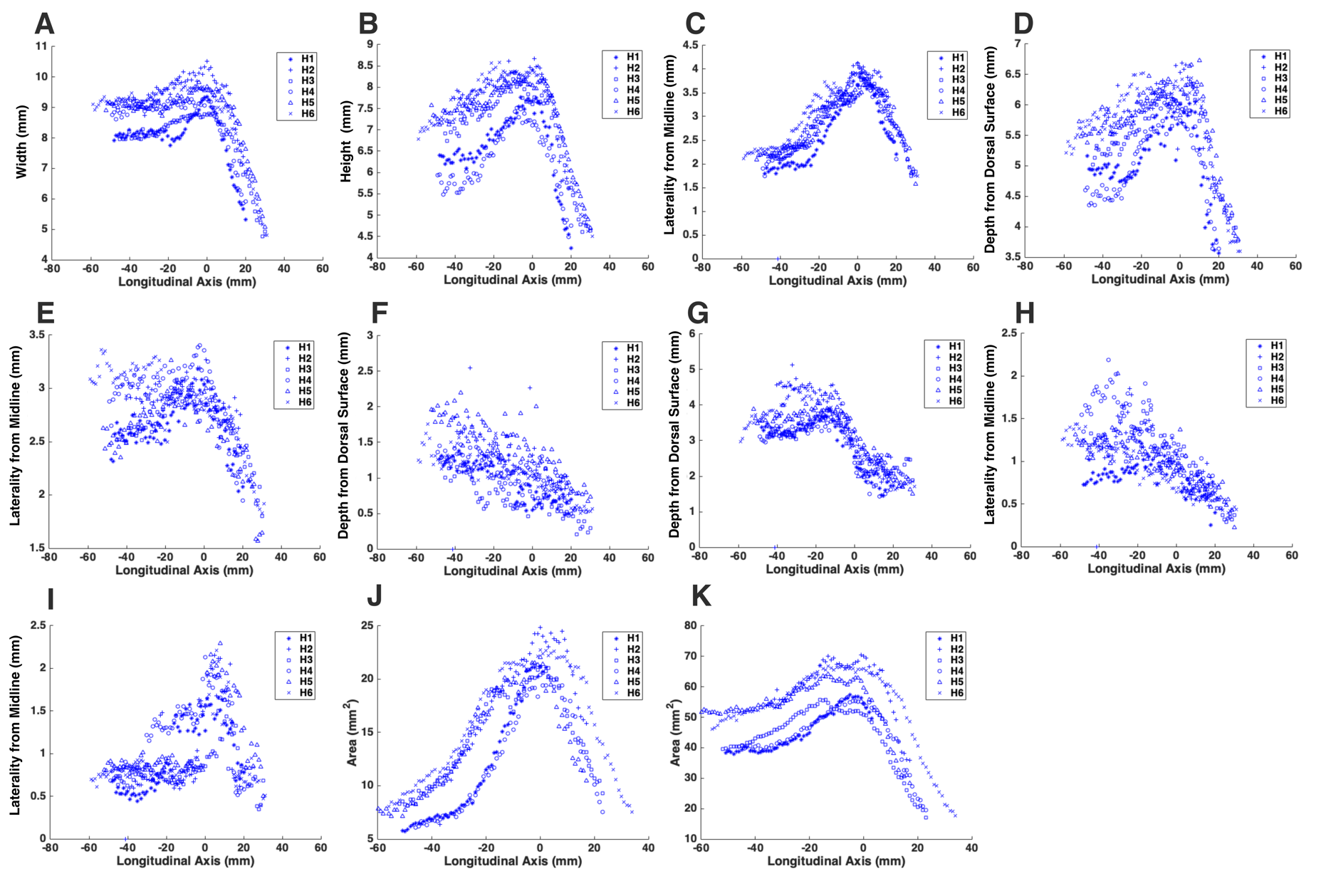


**Fig. S5.** Cross-sectional dimensions of the spinal cord and locations of the ventral and dorsal horns of human cords. Presented data are based on n=6 specimens per species. Different symbols represent different specimens within a species. Measurements are of the parameters shown in Fig. 7 across the length of the lumbosacral cord. All curves have been translated across the x-axis such that the cord’s PCS (where d1 is maximum) is at the origin (x=0). A) d1. B) d2. C) d3. D) d4. E) d5. F) d6. G) d7. H) d8. I) d9. J) a1. K) a2.


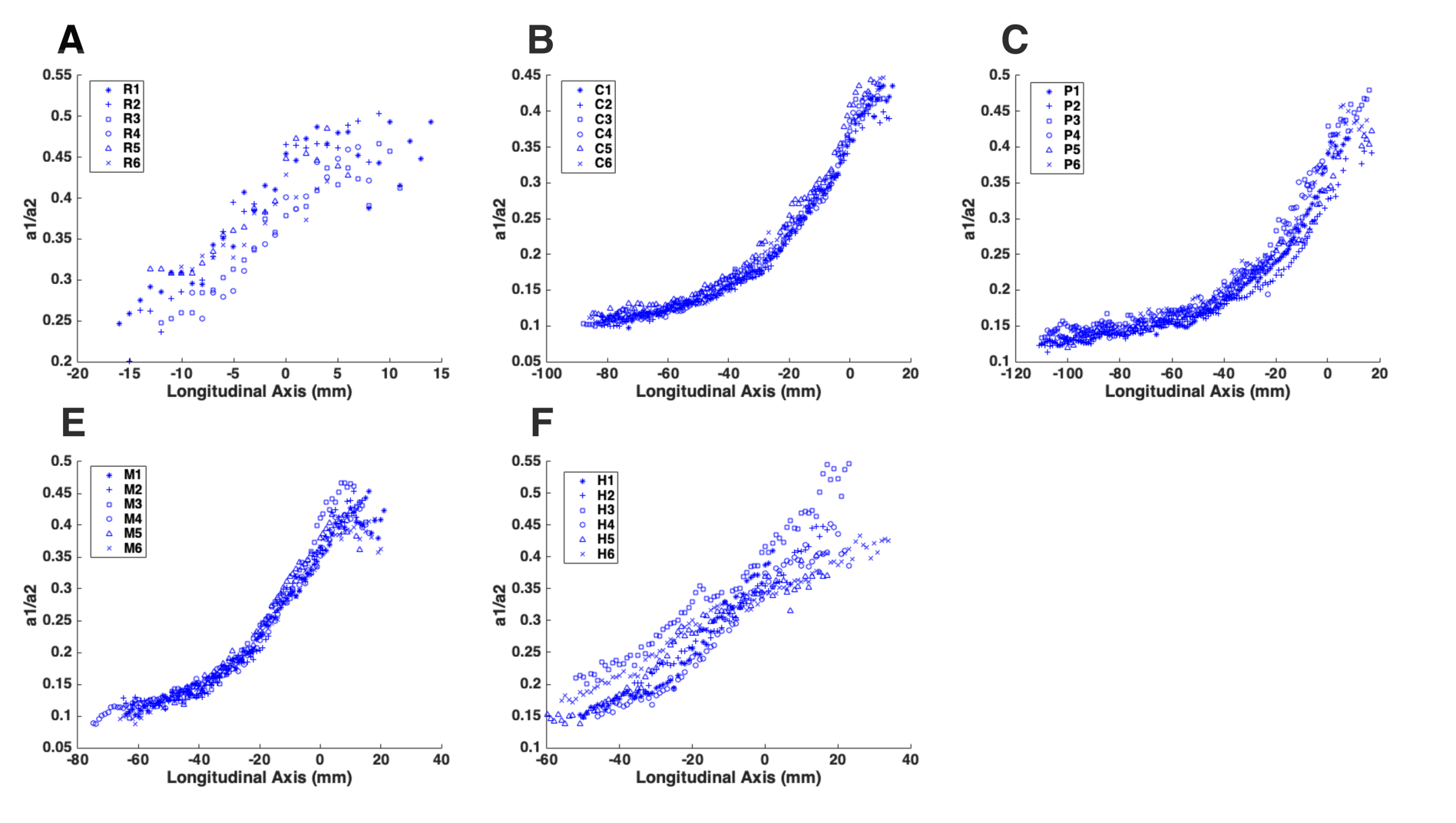


**Fig. S6.** Ratio of the total area of the gray matter to that of the white matter (a1/a2) in all spinal cords of A) Rat, B) Cat, C) Pig, D) Monkey, E) Human. Presented data are based on n=6 extracted specimens per species. Different symbols represent different specimens. All curves have been translated across the x-axis so that each cord’s PCS (where d1 is maximum) is at the origin (x=0)

.
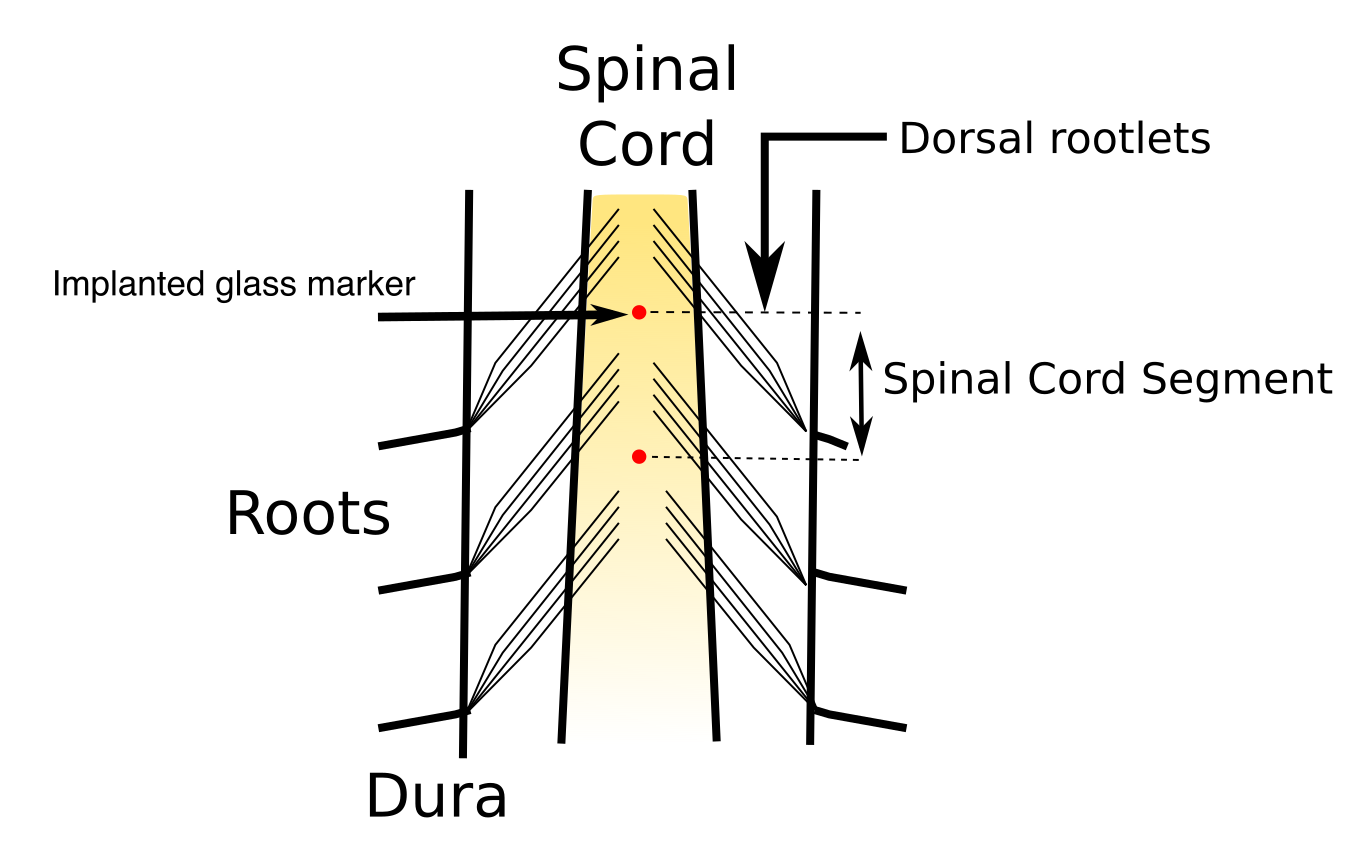


**Fig. S7**. Spinal cord segment identification method (dorsal view). Spinal cord segments were identified based on the location of dorsal rootlets.
